# Editorial: Translational research in hepatitis E, Vol II

**DOI:** 10.3389/fmicb.2022.1011779

**Published:** 2022-08-24

**Authors:** Anna Kramvis, Maria Teresa Pérez-Gracia, Kavita Satish Lole, Manuel Rodriguez-Iglesias, None Shalimar

**Affiliations:** ^1^Hepatitis Virus Diversity Research Unit, School of Clinical Medicine, Faculty of Health Sciences, University of the Witwatersrand, Johannesburg, South Africa; ^2^Departamento de Farmacia, Universidad Cardenal Herrera-CEU, CEU Universities, Valencia, Spain; ^3^National Institute of Virology, Indian Council of Medical Research (ICMR), Pune, India; ^4^Department Microbiology, Hospital Universitario Puerta del Mar, Cadiz, Spain; ^5^All India Institute of Medical Sciences, New Delhi, India

**Keywords:** HEV, epidemiology, pathogenesis, diagnostics, treatment, vaccines

This second volume of translation research in hepatitis E has demonstrated that HEV research is active throughout the world including in Belgium, China, Egypt, India, Spain and the USA. The interest in this research is clear from the high number of views of all articles in this volume.

This second volume contains one review and five original research articles covering a wide range of areas including animal models, diagnosis, molecular virology, treatment, and zoonotic infection.

Hepatitis E virus (HEV) causes about 14 million infections with 300,000 deaths and 5,200 stillbirths worldwide annually and is the most common cause of acute hepatitis. Besides infecting the liver, HEV has extrahepatic tropism. The result of the study carried out by Sayed et al., confirm and expand previous findings showing the persistence and replication of HEV in peripheral blood mononuclear cells (PBMCs). Thus implicating PBMCs as a reservoir for HEV, a potential source for infection for extrahepatic targets and for recurrent HEV infection and the development of chronicity, especially in the settings of immunosuppression. Despite the limitation of the small number of samples, for the first time, the authors report HEV replication in the PBMCs of acute hepatitis patients. Positive and/or negative HEV RNA strands and HEV protein were detected in the PBMCs of acute hepatitis patients. HEV replication in PBMCs was associated with the induction of innate and adaptive immune response, being more pronounced in the PBMCs with the detectable (–) RNA strand.

Zoonotic infection and cross-species transmission are important for the survival and persistence of viruses in nature. HEV has been detected in a variety of domestic and wild mammals and is transmitted to humans mainly by the consumption of raw or undercooked meat or organs. In their study, Rivero-Juarez et al. detected HEV RNA in 37.9% wild boars and in 34.5% *Hyalomma lusitanicum* ticks feeding on wild boars. The prevalence of HEV in wild-boars was relatively high, coinciding with earlier findings in the same geographical area and sampled in autumn (October–November). This is the first time that HEV RNA was detected in ticks, implicating them as a possible host of HEV and playing a role in HEV transmission. However, as correctly stated by the authors, more studies are necessary to evaluate the possibility of maintenance and transmission of HEV through ticks.

In order to study any viral infection various *in vitro* and *in vivo* models are necessary. The lack of an efficient small animal model has hampered the study of HEV and the discovery of anti-HEV therapies. Human liver-chimeric mice have greatly aided in the understanding of HEV, however, the immunodeficient nature of this model prevents the study of all aspects of virus-host interaction. In order to overcome this limitation, non-humanized mice have been used. Collignon et al. compared the susceptibility of human liver-chimeric and non-humanized mice to a pig-derived HEV-4 strain (BeSW67HEV4-2008). They showed that humanized mice could be readily infected with this isolate, extending studies on HEV-1 and HEV-3 strains. On the other hand, in contrast to other studies with other strains of HEV-4, non-humanized mice were not susceptible to the HEV-4 strain used. As the HEV-4 strains belonged to different subtypes, this intimates different infection capabilities of HEV-4 subtypes and requires further investigation and has implications for the relevance of HEV subtypes on host susceptibility and pathogenesis.

Without specific biomarkers for drug-induced liver injury (DILI), DILI has been diagnosed by the exclusion of other causes of liver dysfunction and identification of potential causative drugs. However, as HEV diagnosis is unavailable in many resource-limited settings it is not included in the exclusion criteria. In order to determine whether the absence of HEV testing led to the inadvertent diagnosis of DILI, El-Mokhtar et al. retrospectively analyzed 80 plasma suspected DILI samples for HEV markers. Recent or ongoing HEV infection was identified in 15% of the samples. The level of liver enzymes such as alanine transaminase (ALT) and aspartate transaminase (AST), but not alkaline phosphatase (ALP), was significantly higher in HEV confirmed cases compared to non-HEV confirmed cases. ALT level of at least 415.5 U/L, AST at least 332 U/L, or R-value of at least 5.08 could categorize the putative DILI patients to be tested for HEV infection. Thus as has been previously shown in high-income countries in individuals infected with HEV genotype 3, in a resource-limited setting, liver function tests can be used to differentiate DILI from acute HEV genotype 1 infection, with high sensitivity and acceptable sensitivity.

Zinc has been known to have antiviral activity and the ability to boost the immune response. However, the body absorbs only 10–20% of the ingested zinc and increasing the levels of zinc intake may be cytotoxic. Thus the route of administration of zinc and its bio-availability are important in ensuring its therapeutic benefit. Gupta et al. synthesized ZnO nanoparticles [ZnO(NP)] and tetrapod-shaped ZnO nanoparticles [ZnO(TP)] and evaluated their antiviral activity against HEV and HCV. Both ZnO(NP) and ZnO(TP) were found to be noncytotoxic to cells, as demonstrated by measurement of cell viability and intracellular reactive oxygen species levels. ZnO nanoparticles and tetrapods were found to act independently of the viral entry step, inhibiting viral replication. Tetrapods were found to be superior in their antiviral activity compared to nanoparticles.

Hepatitis E virus (HEV) is a small icosahedral positive-sense, single-stranded RNA virus. The HEV genome includes three open reading frames (ORF1–3). ORF2 encodes the structural capsid protein, which is involved in the viral entry and modulation of the host immune response Zhou et al. review the diverse roles of HEV-ORF2 protein. In addition to making up the capsid, HEV-ORF2 protein exists as different forms in the infected cell, including the secreted form of the full-length ORF2 and cleaved forms of ORF2 proteins. ORF2 protein has an endoplasmic reticulum (ER)-directing signal peptide on its N-terminus. Currently, available literature demonstrates that HEV-ORF2 can be translated into different forms that undergo various post-translational processing and perform different functions, such as host innate immune response and cell signaling regulation, determination of host tropism, and participation in HEV pathogenesis. Understanding the molecular biology of the ORF2 protein will allow for deeper understanding of the replication of HEV and how this can be prevented leading to control and/or cure.

All the contributors to this second volume of *Translational Research in Hepatitis E* have generated knowledge in many aspects of HEV infection. This knowledge has the potential to aid in the design of preventative and treatment modalities necessary to counter HEV infection.

## Author contributions

AK wrote the original draft, which was reviewed, revised and accepted by all Research Topic editors MTP-G, KL, MR-I, and NS. All authors contributed to the article and approved the submitted version.

## Conflict of interest

The authors declare that the research was conducted in the absence of any commercial or financial relationships that could be construed as a potential conflict of interest.

## Publisher's note

All claims expressed in this article are solely those of the authors and do not necessarily represent those of their affiliated organizations, or those of the publisher, the editors and the reviewers. Any product that may be evaluated in this article, or claim that may be made by its manufacturer, is not guaranteed or endorsed by the publisher.

